# Polynuclear ruthenium organometallic compounds induce DNA damage in human cells identified by the nucleotide excision repair factor XPC

**DOI:** 10.1042/BSR20190378

**Published:** 2019-07-16

**Authors:** Olivia G. Fast, Brittany Gentry, Liah Strouth, Madison B. Niece, Floyd A. Beckford, Steven M. Shell

**Affiliations:** Department of Natural Sciences, The University of Virginia’s College at Wise, 1 College Avenue, Wise, VA, 24293

**Keywords:** chemotherapy, cytotoxicity, nucleotide excision repairn, ruthenium, XPA, XPC

## Abstract

Ruthenium organometallic compounds represent an attractive avenue in developing alternatives to platinum-based chemotherapeutic agents. While evidence has been presented indicating ruthenium-based compounds interact with isolated DNA *in vitro*, it is unclear what effect these compounds exert in cells. Moreover, the antibiotic efficacy of polynuclear ruthenium organometallic compounds remains uncertain. In the present study, we report that exposure to polynuclear ruthenium organometallic compounds induces recruitment of damaged DNA sensing protein *Xeroderma pigmentosum* Group C into chromatin-immobilized foci. Additionally, we observed one of the tested polynuclear ruthenium organometallic compounds displayed increased cytotoxicity against human cells deficient in nucleotide excision repair (NER). Taken together, these results suggest that polynuclear ruthenium organometallic compounds induce DNA damage in cells, and that cellular resistance to these compounds may be influenced by the NER DNA repair phenotype of the cells.

## Introduction

Ruthenium compounds are an attractive and actively researched alternative to platinum-based chemotherapeutics. Previous studies indicate a wide range of potential biological activities of ruthenium-based compounds, including anti-tumor cell and anti-viral properties [1–3]. These biological properties are dependent on a number of factors, such as the number of metal centers and the structural organization of the organic substituent groups [4–12]. However, to date, much remains to be determined regarding the activities of polynuclear ruthenium organometallic compounds in cells and what cellular responses are elicited.

**Figure 1:**
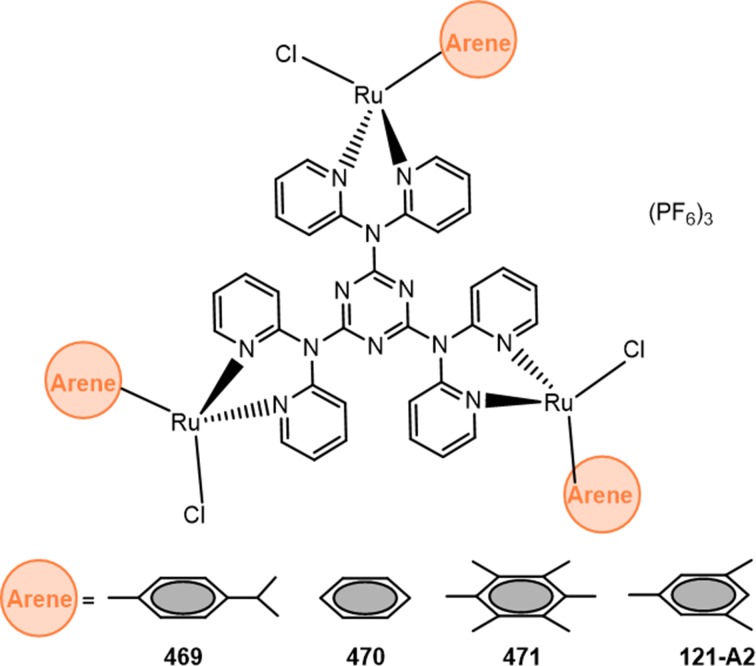
Structures of the polynuclear ruthenium organometallic compounds

As part of a study, looking at the development of ruthenium cage molecules based on a 2,4,6-tris(di-2-pyridylamino)-1,3,5-triazine ligand system (Figure 1), we reported on the synthesis of star-shaped trinuclear complexes [13]. These polynuclear ruthenium organometallic compounds were shown to interact with DNA *in vitro*, and are also effective in binding to human proteins [14]. We had hypothesized that in a mechanism similar to what is usually proposed for mononuclear compounds, the Ru-chloride bond would hydrolyze *in vitro* to provide a reactive intermediate with a Ru-H_2_O bond [15–17]. This intermediate would then covalently bind to DNA molecules generating single-stranded adducts. These properties suggest the potential of polynuclear ruthenium organometallic compounds to form bulky covalent DNA lesions. However, these compounds displayed underwhelming cytotoxic effects when evaluated against a triple-negative breast cancer cell line [13].

Nucleotide excision repair (NER) is a versatile, error-free mechanism to identify and remove a wide assortment of chemically unrelated lesions [18]. NER can be classified into two sub-pathways based on the way DNA lesions are identified [19]. In transcription coupled NER (TC-NER), damaged DNA is identified by the stalling of RNA polymerases when they encounter bulky covalent DNA lesions [20]. In global genome NER (GG-NER), damaged DNA is identified through the DNA-binding activity of the protein *Xeroderma pigmentosum* Group C (XPC), which serves as a general sensor of damaged DNA [21,22]. Both TC-NER and GG-NER funnel into a common biochemical mechanism to excise the damaged DNA strand [23,24]. The excision process is coordinated by the XPA protein, which organizes the nucleases on the DNA substrate. Although XPA possesses no enzymatic activity, it is absolutely required for the NER process [25–27].

Many transition metal chemotherapeutics, such as cisplatin, work by inducing bulky forms of covalent DNA damage in tumor cells [28,29]. As these types of DNA damage are repaired by the NER pathways [30–34], it is possible that NER activity is important in determining the cytotoxic effects of polynuclear ruthenium organometallic compounds. We hypothesized that cellular exposure to these compounds may result in DNA damage that is repaired by NER. Our results demonstrate that exposure to polynuclear ruthenium organometallic compounds induces accumulation of the damaged DNA sensor protein XPC into chromatin-immobilized nuclear foci. Moreover, our results indicate that loss of NER activity sensitized cells to one of the ruthenium compounds tested. These results suggest that polynuclear ruthenium organometallic complexes induce DNA damage in cells which may be repaired by the NER system.

## Materials and methods

### Reagents

Polynuclear ruthenium compounds (Figure 1) were prepared as described previously [13] and dissolved in DMSO. 1× Dulbecco’s modified eagle medium (1x DMEM) and fetal bovine serum (FBS) were obtained from Gibco. Dulbecco’s phosphate buffered saline (1x DPBS) was prepared from molecular biology grade reagents and sterilized by autoclave. MTT [3-(4,5-Dimethylthiazol-2-yl)-2,5-diphenyltetrazolium bromide] was obtained from Calbiochem. Cisplatin was obtained from Strem Chemical. Etoposide was obtained from Millipore Sigma. N,N-dimethylformamide (DMF), sodium dodecyl sulfate (SDS), and glacial acetic acid were purchased from Fisher Scientific. Mouse-anti-XPC (3.26) was obtained from Novus Biologicals. Rabbit-anti-Phospho-H2AX (Ser^139^) was obtained from Bethyl Laboratories. Secondary antibodies Alexa Fluor 488 goat-anti-mouse, Alexa Fluor 568 Donkey-anti-rabbit, and ProLong antifade were purchased from ThermoFisher Scientific.

### Cell culture

HeLa cells were obtained as a generous gift from Professor Yue Zou, East Tennessee State University. XPC^−/−^ (GM16093) and XPA^−/−^ (GM04429) cells were obtained from Coriell Cell Repositories. Cells were cultured in 1x DMEM, supplemented with 10% FBS, and passaged according to supplier’s instructions. Cultures were incubated under standard growth conditions at 37°C, 5% CO_2_.

### Cell viability assays

Cell viability was monitored via MTT assay. Cells were harvested by trypsinization and seeded into 96-well tissue culture-treated microtiter plates. Cells were seeded at a density of 20,000 cells/ml and allowed to adhere for 12 h post harvest under standard growth conditions. Compound solutions or DMSO (mock-treatment) were added at a 1:100 dilution into each well and incubated under standard growth conditions for up to 48 h post addition. MTT (5 g/l in 1× DPBS) was added at a 1:10 dilution to each well and incubated for 4 h under standard conditions. An equal volume of Solubilization Buffer [40% (v/v) DMF, 26% (w/v) SDS, 2% (v/v) acetic acid, pH 4.7] was added and incubated under standard conditions for 10 min. Absorbance was measured at λ570 nm using a BioTek Cytation1 Multi-Mode plate reader. Percent change in growth was determined relative to mock-treated cultures and analyzed using ANOVA and a one-tailed *t* test.

### Chronic exposure assay

HeLa cells were seeded into individual 25 cm^2^ T-flasks at a density ∼100,000 cells per ml culture. Cells were grown in 1× DMEM or 1× DMEM supplemented with either DMSO (1:100 v/v) or ruthenium compound (1:100, 50 μM final concentration). Cultures treated with cisplatin or etoposide (5 μM final concentration) served as positive controls. Cultures were observed daily for 14 days and allowed to grow until ∼80–90% confluence. Cultures were split using a standard 1:5 ratio; compound was added to the indicated concentration after each split. Culture collapse was defined as the point where no viable cells remained on the growth surface of the culture flask.

### Immunofluorescence microscopy

The nuclear focus formation assay was performed as described previously [35–38]. Cells were seeded onto 22 mm^2^ sterile glass coverslips at a density of 20,000 cells/ml and allowed to adhere for 12 h post harvest under standard growth conditions. Compound solutions or DMSO (mock-treatment) were added at a 1:100 dilution and cultures incubated under standard growth conditions for 24 h post addition. Cultures were washed three times with ice-cold 1× DPBS, then incubated in nuclear extraction buffer (1× DPBS + 0.5% (v/v) NP-40) for 5 min on ice to remove proteins not in stable complex with the chromatin. Nuclei were fixed by incubation with methanol:acetone (1:1 v/v) at −20°C for 10 min. Sample were blocked in 1× DPBS + 15% (v/v) FBS). Samples were stained with primary antibodies mouse-anti-XPC (1:200) and rabbit-anti-phospho-H2AX[Ser^139^] (1:200), and secondary antibodies (1:250). Coverslips were mounted in ProLong antifade medium. Fluorescence micrographs were acquired using a BioTek Cytation1 Multi-Mode plate reader equipped with a 40× magnification objective and λ_max_EX 469 nm/λ_max_EM 525 nm (green) and λ_max_EX 531 nm/λ_max_EM 647 nm (red) filter sets. The percentage of cells with greater than 3 foci per nucleus was determined. Samples were coded, with data acquisition and analysis performed using single-blind protocols.

## Results

### Polynuclear ruthenium organometallic compounds induce XPC nuclear foci

Previous studies suggest that polynuclear ruthenium organometallic compounds interact with DNA *in vitro* via a number of mechanisms, including groove binding [39,40]. We had hypothesized that these compounds may form an intermediate that would then covalently bind to DNA molecules to generate single-stranded adducts [15–17]. The XPC protein serves as a general sensor of damaged DNA, exquisitely sensitive in identifying the presence of multiple classes of DNA lesions throughout the genome [33]. Therefore, we hypothesized that if the polynuclear ruthenium compounds behave as expected, exposing cells to these compounds would stimulate recruitment of XPC to the chromatin. To test our hypothesis, immunofluorescence microscopy was used to evaluate formation of chromatin-immobilized XPC in HeLa cells.

**Figure 2:**
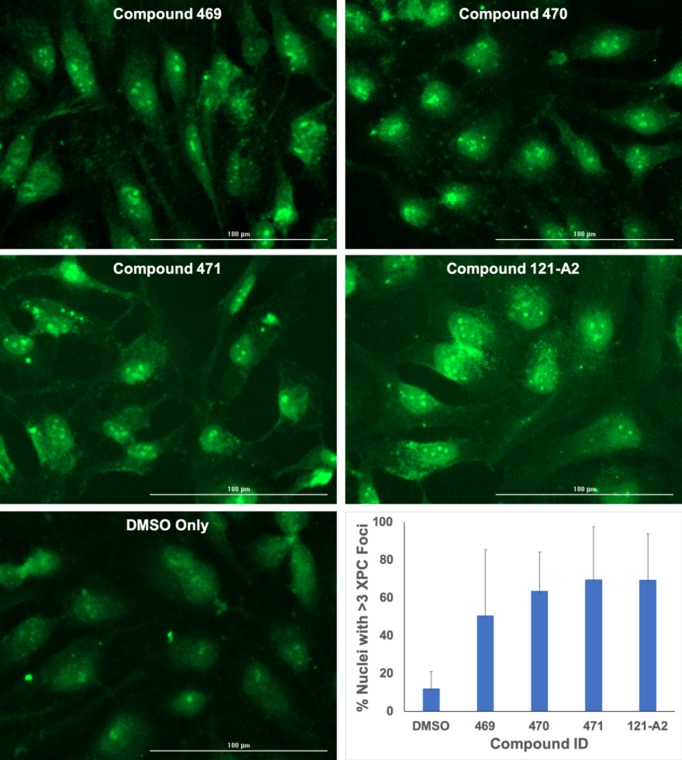
Ruthenium compounds induce XPC nuclear focus formation HeLa cells were treated with compounds (10 μM) or DMSO for 24 h under standard growth conditions. XPC foci were visualized by immunofluorescence microscopy and quantified as the percent of nuclei containing three or more distinct XPC foci. Quantification illustrates the mean of three independent experiments.

As shown in Figure 2, exposure to any of the four polynuclear ruthenium organometallic compounds resulted in multiple XPC foci within the nuclei compared with the mock-treated cells. We quantified the number of cells incurring DNA damage as the percentage of the total population of nuclei containing XPC foci. Analysis of the DMSO-treated cells indicated that most nuclei contain at least one discrete XPC focus. However, only ∼10% of DMSO treated nuclei contain three or more XPC foci. These results suggest that nuclei with less than three XPC foci represent the baseline DNA damage load due to endogenous genotoxins and exposure to the DMSO solvent. We next determined the percentage of HeLa nuclei with three or more XPC foci following treatment with the four ruthenium compounds. Exposure to any of the four compounds increased the number of nuclei with three or more XPC foci relative to mock-treated cells (Figure 2). Replicate experiments support the observed trend that exposure to the ruthenium compounds results in an average of a five-fold increase in the percentage of nuclei with three or more XPC foci. These results suggest that exposure to the polynuclear ruthenium compounds induce DNA damage in cells that is identified by the XPC DNA damage sensor protein.

**Figure 3:**
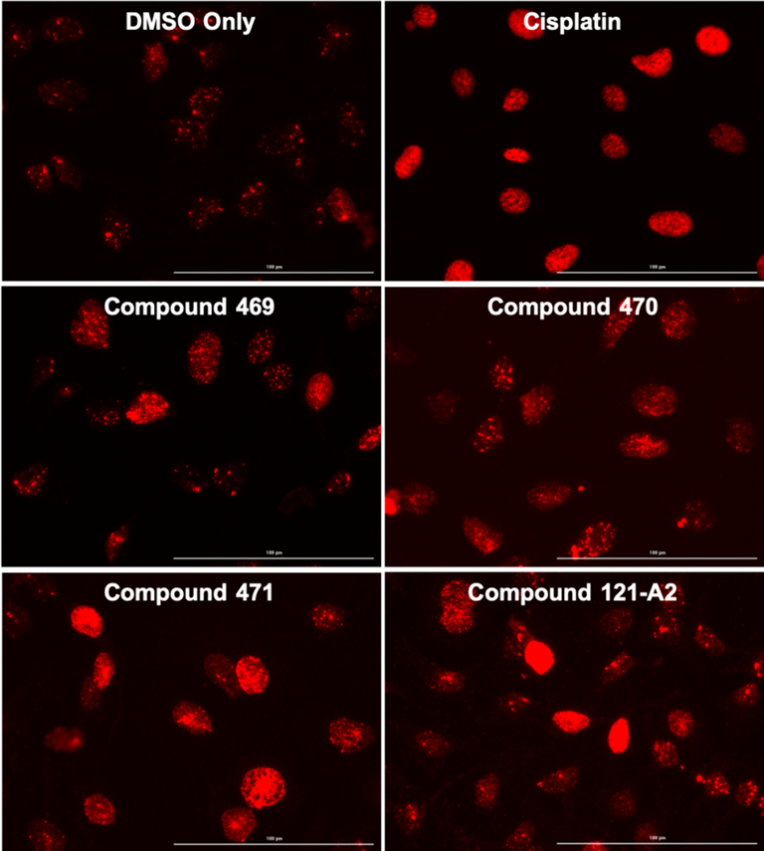
Ruthenium compounds induce phosphorylation of histone H2AX HeLa cells were treated with cisplatin (10 μM), ruthenium compounds (10 μM), or DMSO for 24 h under standard growth conditions. Phosphorylation of H2AX was monitored by immunofluorescence microscopy.

In order to verify the increased retention of XPC protein in the chromatin was the result of DNA damage events, we next observed the phosphorylation of histone H2AX by immunofluorescence microscopy. H2AX phosphorylation is a common marker of an active DNA damage response mediated by the ATR/ATM kinase cascade and activation of the NER process [41–43]. As seen in Figure 3, HeLa cells incubated with DMSO only display diffuse staining intensity for phosphorylated H2AX, similar to previously published studies [41,44,45]. Incubation with 10 μM cisplatin results in an overall increase in both the staining density and intensity for phosphorylated H2AX, indicative of a DNA damage response. Treatment with any of the four ruthenium complexes also resulted in an increase in staining density and intensity for phosphorylated H2AX relative to the mock-treated cells. Taken together with the XPC focus formation results, these results support the hypothesis that exposure to polynuclear ruthenium compounds results in DNA damage that elicits a DNA damage response in human cells.

### Cytotoxicity in NER-compromised cell lines

**Figure 4:**
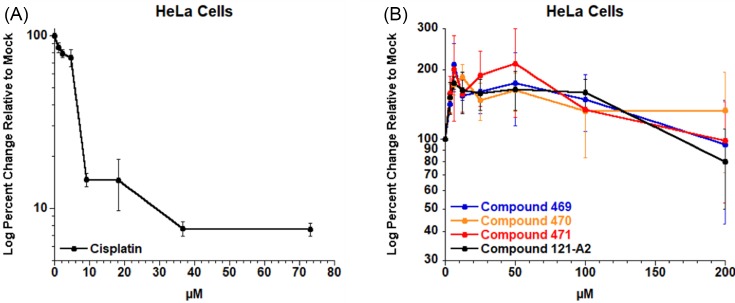
HeLa cell viability assays HeLa cells were incubated with indicated concentrations of cisplatin (**A**) or ruthenium compounds (**B**) for 48 h under standard growth conditions. Cell viability was measured using MTT and analyzed as percent change in growth relative to mock-treated cells.

Minor cytotoxic effects were observed for the compounds under investigation in a triple-negative breast cancer cell line compared with control cells, and were observed only for very high doses [13]. We chose to validate these results using a different cell line. HeLa cells were chosen, as they have highly active NER capacity and have been used extensively to determine repair activity against multiple distinct classes of DNA lesions [46–50]. Cell plating density was optimized to ensure strong signal in the MTT assay and to ensure actively-replicating cell cultures through the experiment. Cisplatin, which induces both bulky covalent lesions and interstrand DNA crosslink damage and is included as a positive control. As expected, cisplatin displayed strong cytotoxicity against the HeLa cell line (Figure 4A). However, none of the four ruthenium compounds displayed significant cytotoxicity in HeLa cells when incubated for up to 48 h after dosing (Figure 4B). These results corroborate the previous report [13] of low cytotoxicity associated with these polynuclear ruthenium organometallic compounds and suggest that cell type does not significantly influence cytotoxicity.

As exposure to these compounds appears to induce DNA damage identified by the XPC protein (Figure 2), we sought to determine whether the apparent lack of cytotoxicity was influenced by NER activity. Therefore, we next tested the cytotoxic properties of the four polynuclear ruthenium organometallic compounds against cells lines with compromised NER phenotypes. We selected two cell lines derived from *Xeroderma pigmentosum* patients to test the influence of NER activity on cytotoxicity. GM16093 cells do not express the XPC protein, which is required for GG-NER, but is not required for the TC-NER pathway, and thus have reduced NER activity. GM04429 cells do not express the XPA protein necessary for both the GG-NER and TC-NER pathways, and thus have no NER activity [19,32].

**Figure 5:**
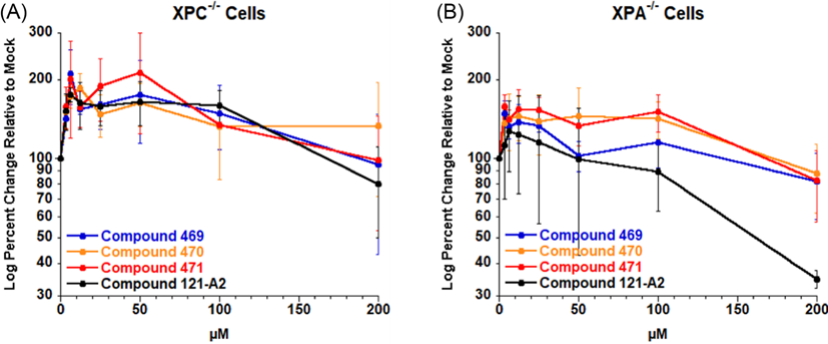
Effects of NER phenotype on cell viability XPC^−/−^ (**A**) and XPA^−/−^ (**B**) cells were incubated with indicated amounts of ruthenium compounds for 48 h under standard growth conditions. Cell viability was measured using MTT and analyzed as percent change in growth relative to mock-treated cells.

Similar to the results for the HeLa cells, none of the four polynuclear ruthenium compounds display significant cytotoxic effects in the XPC-deficient cell lines after 48 h of exposure (Figure 5A). These results were quite unexpected given that all four compounds stimulated recruitment of XPC into chromatin-immobilized foci (Figure 2). We next tested cytotoxicity in the XPA-deficient cell lines. Three of the four compounds (469, 470, and 471) demonstrated no significant cytotoxicity effects after 48 h post exposure in XPA^−/−^ (Figure 5B) cell lines. However, compound 121-A2 did reduce cell viability in the XPA^−/−^. We were able to estimate a lethal dose 50 (LD_50_) value of ∼150 μM for ruthenium compound 121-A2.

**Figure 6:**
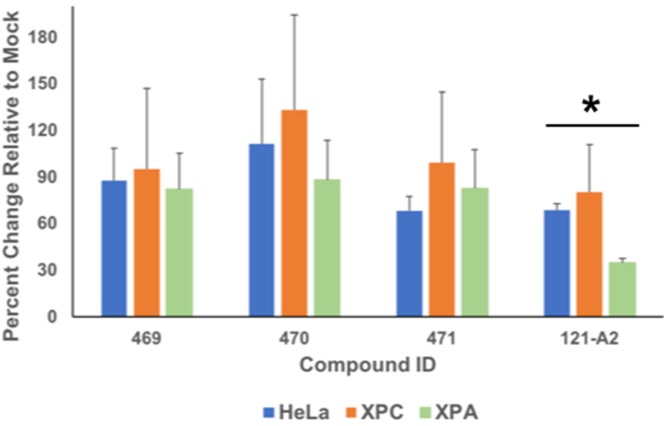
NER phenotype influences cytotoxicity of polynuclear ruthenium compounds Cell viability was compared for HeLa, XPC^−/−^, and XPA^−/−^ cells treated with 200 μM polynuclear ruthenium organometallic compounds. * indicates ANOVA *P*<0.05.

Although the effects were only observed at relatively high doses (>100 μM), the enhanced toxicity for compound 121-A2 is reproducible. In order to evaluate whether NER activity is a sensitizing factor, we compared the cytotoxicity in the three cell lines tested for the maximum dose (200 μM) of each compound tested (Figure 6). ANOVA analysis indicated there were not significant differences in the cell viability among the three cell types when treated with compounds 469, 470, or 471. However, we did observe a statistically significant difference (*P*=0.045) in cell viability among the three cell types when treated with compound 121-A2. We then used a one-tailed *t* test to compare the mean change in cell viability in a pair-wise fashion among the three cell lines. There was no statistically significant difference between the HeLa (NER-proficient) and XPC^−/−^ (NER-reduced) cell lines. We did observe a statistically significant difference between HeLa (NER-proficient) and XPA^−/−^ (NER-deficient) (*P*=0.0012) and between XPC^−/−^ (NER-reduced) and XPA^−/−^ (NER-deficient) cell lines (*P*=0.041). These results suggest that NER activity may play a role in the overall mechanism of cytotoxicity for some, but not all, polynuclear ruthenium organometallic compounds.

We next tested the effects of chronic exposure on cell culture viability. HeLa cells were grown in medium supplemented with either DMSO, ruthenium compound, cisplatin, or etoposide (topoisomerase II inhibitor) for up to 14 days. Effects on cell proliferation were determined as a function of cell confluency and culture split times (Table 1). HeLa cells treated with DMSO grew to confluence within 48 h throughout the course of the experiment and displayed no difference compared with the untreated cells. These results suggest that chronic exposure to DMSO at the indicated concentration does not change the normal growth phenotype for HeLa cells over time. As expected, cultures treated with etoposide collapsed within two days while cultures treated with cisplatin collapsed within three days. Cultures treated with ruthenium compounds 469, 470, and 471 displayed no change in growth rate over the course of the experiment. The culture treated with compound 121-A2 displayed similar growth rates to the DMSO-treated and -untreated cells for the first week of the experiment. However, at day 8 of the experiment the 121-A2 culture had not yet achieved confluence. By day 10 the cell density began to decrease, and by day 14 the culture collapsed. Although a limited and strict qualitative analysis of the culture growth rate, these results suggest that prolonged exposure to compound 121-A2 negatively impacts cell viability over time.

**Table 1:** Effects of chronic exposure to ruthenium compounds on HeLa cell growth

	Untreated	DMSO	Etoposide	Cisplatin	469	470	471	121-A2
Day 0	Start	Start	Start	Start	Start	Start	Start	Start
Day 2	Split	Split	Collapse	No split	Split	Split	Split	Split
Day 4	Split	Split		Collapse (Day 3)	Split	Split	Split	Split
Day 6	Split	Split			Split	Split	Split	Split
Day 8	Split	Split			Split	Split	Split	No split
Day 10	Split	Split			Split	Split	Split	No split
Day 12	Split	Split			Split	Split	Split	No split
Day 14	Split	Split			Split	Split	Split	Collapse

## Conclusions

Up-regulation of NER activity can lead to cancer cell resistance to chemotherapeutics (reviewed in [51]; therefore, understanding the role of DNA repair in cytotoxic mechanism is a critical parameter in improving compound efficacy. Polynuclear ruthenium organometallic compounds represent an emerging avenue for chemotherapeutic development and research. In the present study, we have identified that these classes of compounds have the potential to induce DNA damage in living cells. All four compounds stimulated the recruitment and retention of the damaged DNA sensing protein XPC into the chromatin relative to mock treatment and phosphorylation of the histone H2AX. This is the first evidence that exposure to these ruthenium compounds results in DNA damage in cells. It is important to note that while XPC foci indicate the presence of DNA damage, the broad binding specificity of XPC [33,52–57] prohibits the classification of the type of DNA damage. These results are also quite interesting as these compounds bind to, but do not form covalent bonds with, isolated DNA *in vitro*. It is possible the compounds undergo metabolic activation within the cell and/or stimulate the production of secondary toxicants.

Although the overall toxicity of the tested compounds is low, our study indicates that NER activity played a role in mitigating toxicity for compound 121-A2. Sensitivity to compound 121-A2 was only observed when NER activity was completely abolished. However, the relatively high LD_50_ value (∼150 μM) for compound 121-A2 in only one cell type combined with the lack of enhanced toxicity for the other complexes tested in the XPA^−/−^ cell line suggests that NER phenotype may only be one part of a more extensive mechanism involving additional genome maintenance pathways.

The recruitment of XPC into chromatin-immobilized foci and phosphorylation of H2AX is highly suggestive of DNA damage. However, the delay in toxicity beyond the point at which XPC foci are observed suggests multiple genome maintenance pathways may be involved in mitigating effects of the compounds. Previous studies indicate that the broad substrate recognition properties of XPC may allow for stimulation of other DNA repair processes in cells [58]. As such, NER may or may not be the principal repair pathway responsible for repair of these types of DNA damage and may work in conjunction with other processes. Additionally, human DNA polymerase η (Polη, XPV) is an active participant in the NER process, allowing stalled replication forks to bypass certain covalent lesions and allowing for repair after the conclusion of replication [59]. Polη bypasses cisplatin and oxaliplatin adducts and incorporates the correct nucleotide opposite the damaged nucleotide with relatively high efficiency in G*:C base pairs where G* is the platinum adducted nucleotide [60,61]. Therefore, the role of bypass synthesis in cellular resistance to polynuclear ruthenium organometallic compounds cannot be discarded based on the current evidence.

## Abbreviations

DMEM: Dulbecco’s modified eagle medium
DMF: dimethylformamide
DMSO: Dimethylsulfoxide
DPBS: Dulbecco’s phosphate buffered saline
FBS: fetal bovine serum
GG-NER: global genome NER
H2AX: Histone variant 2A
MTT: 3-(4,5-Dimethylthiazol-2-yl)-2,5-diphenyltetrazolium bromide
NER: nucleotide excision repair
SDS: sodium dodecyl sulfate
TC-NER: transcription coupled NER
XP: *Xeroderma pigmentosum*
XPA: *Xeroderma pigmentosum* complementation group A protein
XPC: *Xeroderma pigmentosum* complementation group C protein
